# Case of Neuralgia of the Par Vagum Imitating Hysteria

**Published:** 1829-04-01

**Authors:** 


### Case of Neuralgia of the Par Vagum imitating Hysteria. By M. René Prus, M.D.

It would be difficult to say, with certainty, whether or not the above title should be reversed, and made to stand thus: "Hysteria imitating Neuralgia." Be this as it may, the case is interesting, and the particulars shall be laid before our readers.

*Case.* Rosina, a femme de chambre, aged 30 years, had been brought up in damp and low apartments, and had suffered severely from rheumatism at the early age of twelve years. At the age of 16, she was so much frightened at the approach of the enemy's troops to the capital, that she was seized with the most excruciating pains in the epigastrium, which came on in paroxysms, with intervals of ease. They were often accompanied by vomiting, and by the sensation of a solid body rising from the stomach into the throat. The paroxysm sometimes ended spontaneously by a copious flood of tears. Frequently, when the epigastric pains disappeared, the patient was tormented with pain in the dental and orbital branches of the fifth pair of nerves—but these were almost always confined to one side of the face. For these complaints, which were considered as hysteria, various remedies were employed, without any success. Such was the history given by the patient when she consulted M. Prus, 14 years after the commencement of her sufferings. She said she did not mind the facial pains; but those in the stomach were intolerable, and rendered her life miserable. M. Prus considered the original cause as a moral one—that is, a violent mental emotion, and the phenomena of epigastric pain, vomiting, and the globus, induced him to look on the affection as one of the recurrent branches of the eighth pair. The intermissions and paroxysms suggested the idea of neuralgia, which was strengthened by the pain shifting from the par vagum to the fifth pair, and *vice versa.* At the time of application, the pains were confined to the stomach, and that between the hours of three and half-past seven, p.m. The sulphate of quinine was prescribed during the intermissions, and, in five days, the pains ceased. The patient considered herself radically cured, and was in high spirits; but, on the 22d day afterwards, the most insufferable pain occurred in the course of the infra-orbital nerve. A sinapism was applied to the arm of the same side, soon after which the pain quitted the face, and seated itself in the cubital nerve of the arm sinapismed. This was of momentary duration, and an interval of 15 days of ease succeeded, and then the facial pain recurred with great violence. A blister was now applied beneath each trochanter, with the view of driving the neuralgia to as great a distance as possible from its usual seat. The sciatic nerves of the right side became violently painful, and completely prevented the patient from walking. Three blisters, with intervals of two days between each, were applied along the course of the nerve. The pain then suddenly shifted to the left sciatic nerve, and flying blisters were again applied, with the effect of removing the pain. From this time (the middle of February, 1828) the patient remained free from pain, and from all the symptoms of an hysterical character before described.

We leave this case in the hands of our readers, though we cannot help inclining to the opinion of M. Prus, that the hysterical symptoms were attributable to the affection of the par vagum, and that neuralgia was, after all, the true name and nature of the disease.—*Journal Générale, Janvier*, 1829.

---

XLVI.

